# Synthetic Modifications of Andrographolide Targeting New Potential Anticancer Drug Candidates: A Comprehensive Overview

**DOI:** 10.3390/molecules29122884

**Published:** 2024-06-18

**Authors:** Gatien Messire, Patrick Rollin, Isabelle Gillaizeau, Sabine Berteina-Raboin

**Affiliations:** Institut de Chimie Organique et Analytique (ICOA), Université d’Orléans, UMR-CNRS 7311, BP 6759, rue de Chartres, 45067 Orléans, Cedex 2, France; gatien.messire@univ-orleans.fr (G.M.); patrick.rollin@univ-orleans.fr (P.R.); isabelle.gillaizeau@univ-orleans.fr (I.G.)

**Keywords:** *Andrographis paniculata*, andrographolide, synthetic modifications, oncology, drug candidates

## Abstract

This review collects the synthetic modifications performed on andrographolide, a natural molecule derived from *Andrographis paniculata*, for oncology applications. Various pharmacomodulations were carried out, and the products were tested on different cancer cell lines. The impact of these modifications was analyzed with the aim of mapping the positions essential for activity to facilitate future research in this field. However, this study makes it clear that, in addition to structural modifications of the molecule, which can result in varying degrees of effectiveness in targeting interactions, the lipophilic capacity of the structures obtained through hemisynthesis is of significant importance.

## 1. Introduction

**Figure 1:**
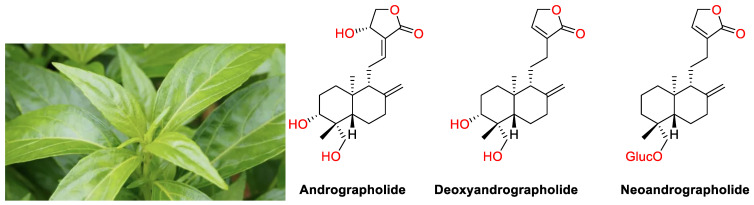
Major compounds found in the leaves of *Andrographis paniculata*.

Andrographolide, a compound found in *Andrographis paniculata,* a plant of the *Echinaceae* family, is a labdane-based diterpenoid. This plant is well known in the Chinese and Ayurvedic pharmacopoeias for its multiple therapeutic applications [1]. Its many activities primarily include the stimulation of the immune system in respiratory pathologies, but also antibacterial, antiviral, anti-inflammatory and anticancer activities, the latter being the focus of this review. The administration of andrographolide regulates internal inflammation by suppressing NF-κB activation, although the mechanisms involved are not yet fully established. Numerous results attest to this activation, as AG suppresses NF-κB activation and COX-2 expression by targeting TBK1 via interactions with TLR3 and TLR4 [2]. These anti-inflammatory properties were also demonstrated in our own studies [3,4,5,6]. Although andrographolide is the diterpene present in the largest amount in *A. paniculata*, we also found diverse analogues in the plant. After extraction, either from the plant or from dietary supplements in tablet or capsule form, according to a described protocol [3], we could show the presence of non-negligible amounts of dehydroxyandrographolide and neo-andrographolide (Figure 1). Other compounds are present, albeit in much smaller quantities.

The aforementioned derivatives also have interesting properties, but the possible synergistic activity of those three main products remains to be demonstrated. Andrographolide is thought to reduce the secretion of several pro-inflammatory cytokines, including IL-1β, IL-6, IL-8, IL-17A and TNF-α, and IFN-γ inhibits NF-κB and ROS production [7,8]. Other precisely described pathways include changes in the PI3K and MAPK pathways and inhibition of NFAT activation [9]. Andrographolide and its derivatives are therefore mainly involved in anti-inflammatory and pro-apoptotic effects. They are therefore thought to limit inflammation [10], which is often the breeding ground for cancer-like cellular disturbances. As andrographolide induces apoptosis in human cells in vitro [11], it appears that synergistic effects with other molecules as well as with molecules within the plant itself are important, since the crude extract has shown more significant effects than pure andrographolide in some studies [12,13]. Despite its low bioavailability, it was shown that andrographolide can cross the blood–brain barrier to reach different levels of the brain and that supplementation can act against cerebral atrophy [14]. This property could also make it an interesting agent against neurodegenerative diseases, since andrographolide could reduce the neuroinflammation [15] often involved in those pathologies. Andrographolide or its pharmacomodulations might bring a solution to other equally important pathologies, but the focus here will be on chemical modifications envisaged in recent years for their biological impact on various cancers.

Although andrographolide and its natural derivatives are undoubtedly bioactive, their main drawback lies in the low content and bioavailability of these molecules, which exhibit poor water solubility [16,17]. Structural and formulation modifications aimed at improving selectivity and bioavailability have been considered [17], but no drug candidates have been developed to date.

In this review, we aim to summarize the synthetic modifications previously performed on andrographolide for oncology applications, with an attempt to understand what makes *A. paniculata* so active. Indeed, andrographolide appears to exhibit cytotoxicity against almost all types of cancer cells, which is why it garners interest [18,19,20,21].

Molecules derived from this plant have been tested on various cancer panels [22,23], including breast [24], colon [25], prostate [26], liver [27], head [28] and other carcinomas. Andrographolide has shown promise in limiting tumor growth by controlling inflammation, particularly in head and neck carcinomas, but also in glioblastomas [29,30]. The presumed mechanisms are numerous but not always easy to identify due to the wide range of potential biological actions.

## 2. Results and Discussion

**Figure 2:**
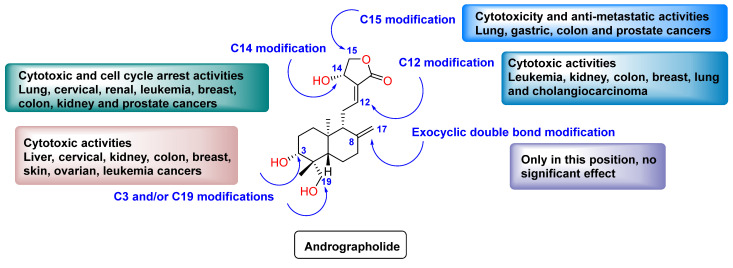
Different structural sites of andrographolide considered for modifications.

The literature reports various modifications of andrographolide to improve its biological activities and bioavailability (or water solubility), mainly on the five-membered lactone ring at the C14 and C15 positions, as well as at C12 and the C3 and C19 alcohol functions of the first six-membered ring. Some additional modifications to the exocyclic double bond have also been addressed. The different pharmacomodulations will be discussed successively in terms of their impact on targeted biological applications and as a function of the andrographolide modification site. The various possibilities are summarized in Figure 2.

### 2.1. Lactone Modification at Position C15

**Scheme 1:**
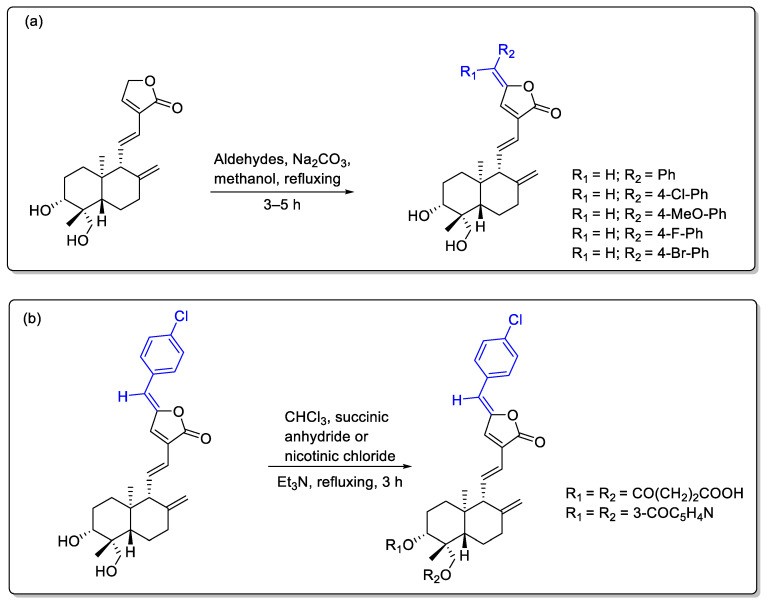
(**a**) Synthesis of C15-benzylidene andrographolide analogues, (**b**) esterification (3,19-disuccinate and 3,19-dinicotinate) on the most active *para*-chlorobenzylidene derivative.

Hepatocellular carcinoma (HCC), an aggressive tumor with a high recurrence rate, is one of the most lethal cancers for which surgical treatment is not often considered. Therefore, it is important to limit cell growth within the tumor and reduce the proliferation of metastases. Andrographolide has been considered for its cytotoxic effects and angiogenesis inhibition activity. Dysfunctional angiogenesis has been associated with the development of several diseases, including cancers [31]. It should be noted that inflammation and angiogenesis are closely linked, making andrographolide a prime raw material for the development of drug candidates [31,32]. Indeed, andrographolide inhibits vascular endothelial growth factor (VEGF)-mediated tumor angiogenesis by binding to vascular endothelial growth factor receptor 2 (VEGFR2), thus exhibiting an antitumor effect [33]. Its inhibitory activity was demonstrated in vivo with a reduction in neoangiogenesis [34,35,36]. Andrographolide derivatives modified at C-14 and/or C-3 and C-19, often by an ester chain, exhibit better cytotoxic activities than andrographolide itself [37,38,39]. Andrographolide has also shown interesting activities against cell migration and invasion in various cancers. Wu et al. aimed to improve andrographolide anti-metastatic activity [40]. Among the modifications carried out, the introduction of a benzylidene at position C15 led to a compound that proved more potent in inhibiting cell migration and showed greater cytotoxicity than natural andrographolide in various cancer cells, such as A549 (lung cancer), SGC-7901 (human gastric cancer), HT-29 (colon adenocarcinoma) and PC-3 (prostate cancer), as shown in Scheme 1a. In general, upon removal of the hydroxyl at C14, inhibition of cell migration increased, while cytotoxicity decreased. On the other hand, the modification of the exocyclic C8–C17 double bond by epoxidation was not effective, resulting in a loss of activity. The same group pursued these investigations by studying the in vivo antitumor activity of those derivatives, particularly against tumor metastasis. Using an orthotopic H22 xenograft mouse model and human umbilical vein endothelial cells (HUVECs) [41], they measured the inhibitory activities, notably, of angiogenesis and hepatoma metastasis, comparing them to those of andrographolide. The results showed that this C15-benzylidene modification significantly improved the anticancer effect in vivo compared with natural andrographolide. These derivatives reduced tumor size and the invasive and metastatic capacities of H22 cells. Although andrographolide and its derivatives are known for their diverse potential activities, this work showed that such a C15-benzylidene-substituted derivative can induce much greater in vivo inhibition of liver cancer growth and metastasis than andrographolide itself.

An additional modification of both C3 and C19 alcohol functions by esterification (3,19-disuccinate and 3,19-dinicotinate) was also tested on the most active *para*-chlorobenzylidene derivative. However, the results were disappointing, showing a loss of inhibitory activity against migration in the tested cell lines, i.e., 5637 (bladder cancer), SGC-7901 (human gastric cancer) and PC-3 (prostate cancer), as shown in Scheme 1b [40].

### 2.2. Lactone Modification at Position C14

**Scheme 2:**
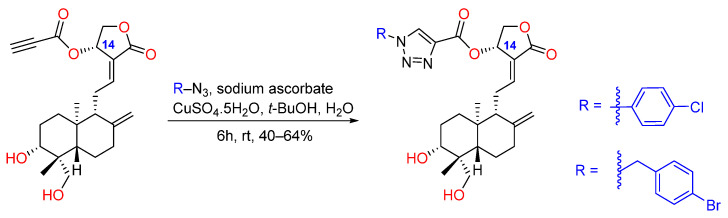
Synthesis of 14α-*O*-(1,4-disubstituted-1,2,3-triazolyl) andrographolide.

Compounds resulting from the C14 esterification reaction of andrographolide play an important role in various medicinal chemistry applications. More recently, some anticancer effects were demonstrated, notably, for derivatives such as 14α-O-(1,4-disubstituted-1,2,3-triazolyl) andrographolide, which exhibit better cytotoxicity than natural andrographolide. These derivatives were obtained from the propargyl ester via Cu(I)-catalyzed click chemistry. Of the compounds synthesized and tested for cytotoxic activity in three cell lines, i.e., HCT-15 (colorectal cancer), HeLa (cervical cancer) and K562 (leukemia), the R = *para*-chlorophenyl-substituted triazole derivative proved more active in HCT-15 cells, whereas the R = *para*-bromobenzyl-substituted triazole compound was the most potent in K562 cells, while maintaining cell viability in the healthy MRC-5 cell line (Scheme 2) [42].

**Scheme 3:**
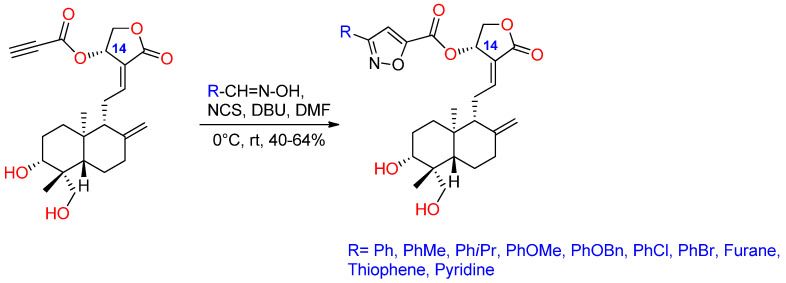
Synthesis of 14α-*O*-(1,4-disubstituted-isoxazolyl) andrographolide.

The same authors also synthesized and evaluated a number of isoxazolyl-based C14 esters of andrographolide (Scheme 3). Their cytotoxic activity was evaluated in three human cancer cell lines, i.e., HCT-15 (colon), HeLa (cervical) and DU-145 (prostate), along with the normal VERO cell line. Overall, these derivatives exhibited enhanced cytotoxicity compared with andrographolide itself and showed no toxicity in the normal VERO control cell line [43]. The authors mentioned further studies in this direction, considering it a promising prospect for the development of drug candidates.

**Figure 3:**
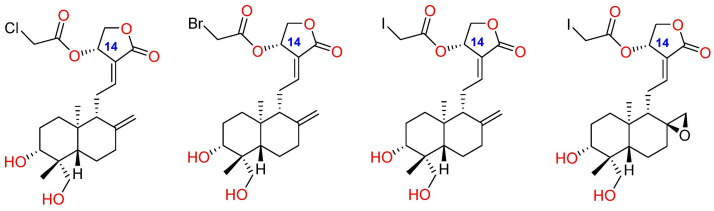
C14 esterified andrographolide analogues.

Some other C14 esters of andrographolide were shown to possess superior anticancer properties compared to andrographolide itself [23]. Among the synthesized esters, the halogenated compounds demonstrated significant cytotoxicity in the MTT cell viability assay in renal (HEK-293) and breast (MCF-7) cancer cells, with low toxicity in normal cells. All tests indicated significant apoptotic properties of these compounds. The brominated and iodinated compounds were the most active in renal and breast cancer cells. The iodo derivative 14-(2-iodoacetyl)-8,17-β-epoxyandrographolide, synthesized via m-CPBA oxidation of a C3–C19 isopropylidene-protected precursor, exhibited similar activity against kidney and breast cancer. The other diastereomeric epoxide was inactive. The authors proposed a mechanism of action involving the activation of p53, leading to NF-κB regulation coupled with the simultaneous regulation of the Bax/Bcl-xl ratio, resulting in apoptosis, thus highlighting these halogenated esters as promising structures (Figure 3).

**Figure 4:**
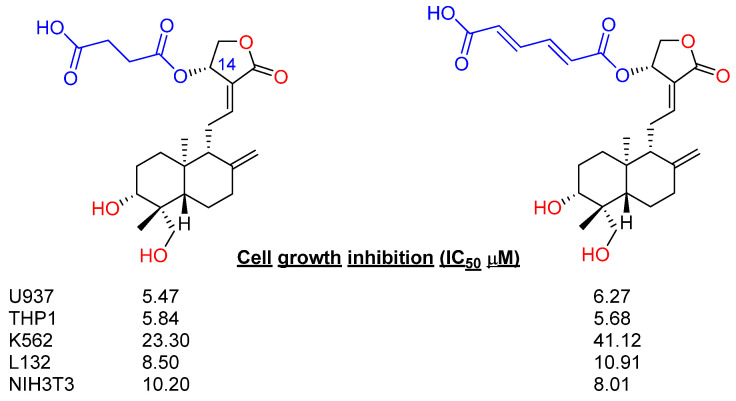
C14 esterified (α-alkylidene-γ-butyrolactone moiety) andrographolide analogues.

Chatterjee et al. [44] focused on the functionalization of the C14 hydroxyl group to assess the in vitro cytotoxicity of the obtained compounds in human leukemia cell lines. Previously, the α-alkylidene-γ-butyrolactone moiety had shown good activity in the micromolar range. However, these products were found to be more cytotoxic, with lower IC50 values in both cancerous and normal cells, compared to andrographolide itself [44] (Figure 4). 

**Figure 5:**
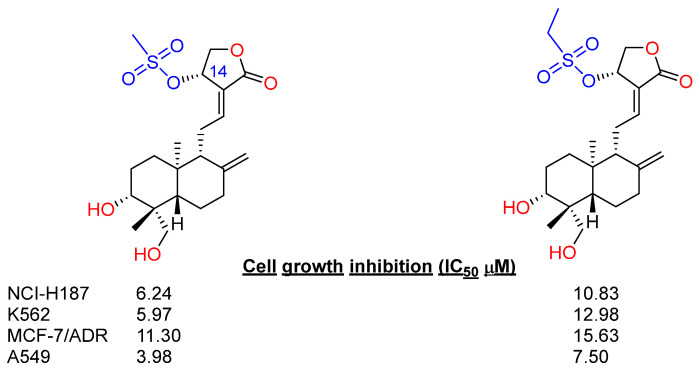
Cytotoxic effects of C-14 sulfonyl ester-substituted analogues in human lung, leukemic, and breast cancer cell lines.

Singh and Agarwal [45] investigated the cytotoxic effects of the C-14 sulfonyl ester substitution in human cell lines of small-cell lung cancer (NCI-H187), leukemia (K562), breast cancer (MCF7/ADR) and lung adenocarcinoma (A549). This molecule showed better cytotoxic activity than andrographolide against NCI-H187, K562 and MCF-7 cell lines [45] (Figure 5).

**Scheme 4:**
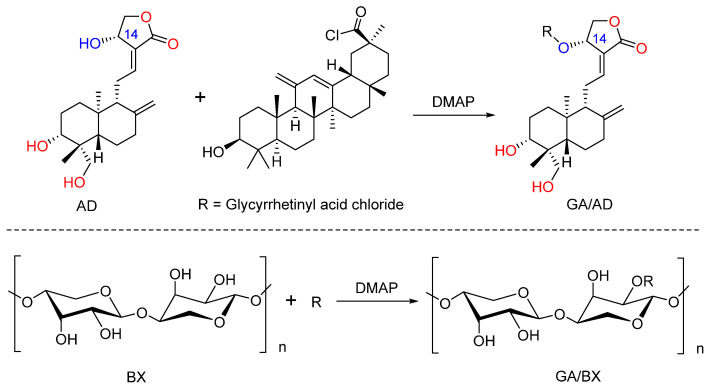
General reaction of the bagasse xylan/andrographolide esterified derivative.

Wang et al. [46] explored the association of plant polysaccharides with andrographolide to enhance the activity of the latter. Plant polysaccharides have been reported to inhibit tumor cell proliferation [47] and increase biological immunity [48]. Xylan could ensure the bioavailability of andrographolide, as it contains numerous hydrophilic hydroxyl groups, which can also be chemically modified. In addition, xylan and its derivatives may possess anticancer activity by promoting cell apoptosis [49,50]. Modifications of andrographolide were designed to reduce hydrophobicity and improve bioavailability. Nanoparticles obtained by combining xylan and curcumin demonstrated a synergistic nanocarrier and cytotoxic effect in human colon cancer [51]. Similarly, the inhibitory and synergistic effects of paclitaxel combined with andrographolide were demonstrated by Yuan et al. [52]. Xylan plays an immunomodulatory role in cancer cell proliferation and differentiation [53,54,55]. Wang et al. [46] used andrographolide and xylan from bagasse, demonstrating an anticancer effect involving different mechanisms of action, generating a potential synergistic effect. A xylan/andrographolide complex was therefore synthesized using a C14 glycyrrhetinyl ester and itaconic acid as a grafting monomer. The introduction of reactive groups by esterification, grafting and cross-linking based on the principle of radical polymerization was envisaged in the form of nanoparticles. Anticancer activity evaluation showed that the inhibition rate of the BX/AD complex, associated with the glycyrrhetinyl ester and itaconic acid, was around 16 times higher in liver cancer cells than that of the BX/AD complex alone. In addition, the combination of BX/AD in the form of esterified nanoparticles improved the solubility and diffusion capacity of the two molecules. Significant inhibitory effects were also demonstrated in gastric and mammary cancer cells but not in normal human liver cells. The anticancer activity of grafted and esterified nanoparticles with xylan/andrographolide was significantly enhanced (Scheme 4).

**Scheme 5:**
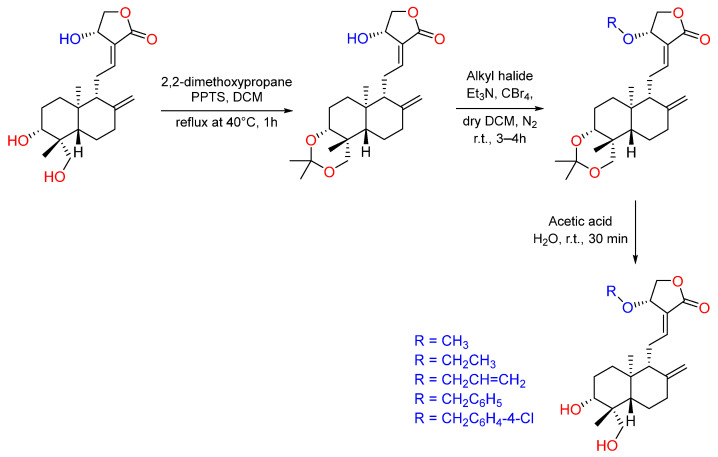
Synthesis of alkoxy andrographolide analogues.

R. Patil et al. [56] reported in 2014 the synthesis of a series of C-14 alkoxy andrographolide derivatives and evaluated their cytotoxic activity against various cancer cell lines, including lung cancer (H522), leukemia (K562), breast cancer (MCF-7/ADR) and prostate cancer (DU145) cell lines. The analogues (Scheme 5) were efficiently synthesized by protecting the C3 and C19 hydroxyls with an isopropylidene ketal, reacting various alkyl halides at the C14 position and performing a final acid-catalyzed deprotection to afford the expected products. The biological cytotoxic activity of andrographolide and its analogues was evaluated in vitro against the mentioned cell lines using the classical MTT method [57]. These modifications improved cytotoxicity to varying degrees compared with that of andrographolide. 

#### 2.2.1. C14, C3 and C19 Modifications

**Figure 6:**
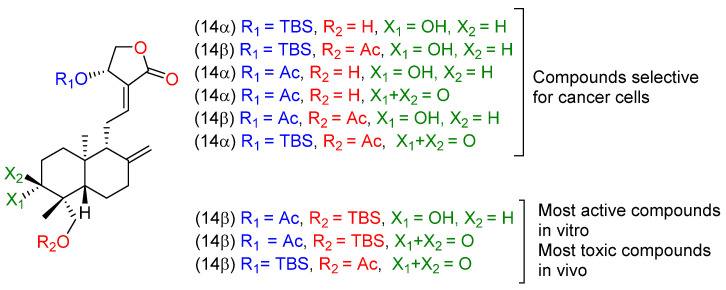
Andrographolide analogues with activity against zebrafish cancer cells.

Zhou et al. [58] conducted a study on the effects of alcohol modifications at positions 14, 3 and 19 of andrographolide on the antiproliferative activity and in vivo toxicity using zebrafish embryos. Their work showed that stereochemistry at position 14, along with alcohol modifications at positions 3, 14 and 19, influenced the proliferative activity and selectivity of the compounds, notably in MDA-MB-231 (triple-negative breast cancer) and A549 (lung cancer) cells. The modifications also affected toxicity to varying degrees. Notably, some isomers, such as the 14α isomers, showed good antiproliferative activity without significant toxicity, except for one compound. In contrast, the 14β isomers exhibited stronger antiproliferative activity but also increased toxicity. Overall, the various substitutions significantly impacted the properties of andrographolide, though no clear generalizations about their efficacy could be made [58] (Figure 6).

**Figure 7:**
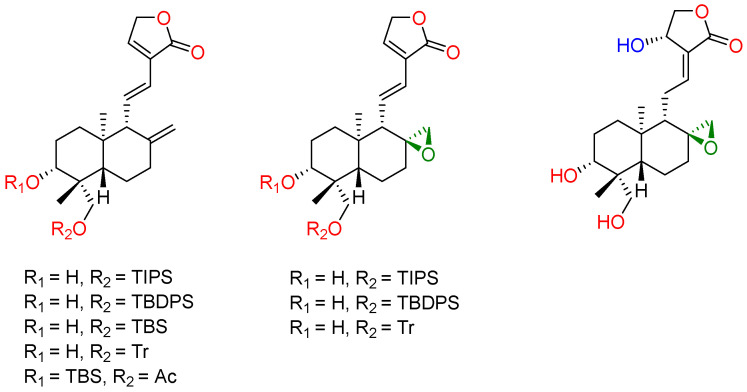
Andrographolide and epoxide analogues with cytotoxic activity.

Saeeng et al. explored the chemical derivatization of the hydroxyl at the C19 position of 14-deoxy-11,12-didehydroandrographolide with silyl, trityl or acetyl groups followed by silylation at the C3 position, as well as the synthesis of C8–C17 epoxides. The epoxidized analogues showed greater inhibition of cholangiocarcinoma (KKU-M213 and KKU-100) than ellipticine, which was used as a positive control. Additionally, these C19 epoxidized and silylated compounds exhibited anticancer activity in micromolar concentrations against the HT-29 colon cancer cell line [59] (Figure 7).

**Figure 8:**
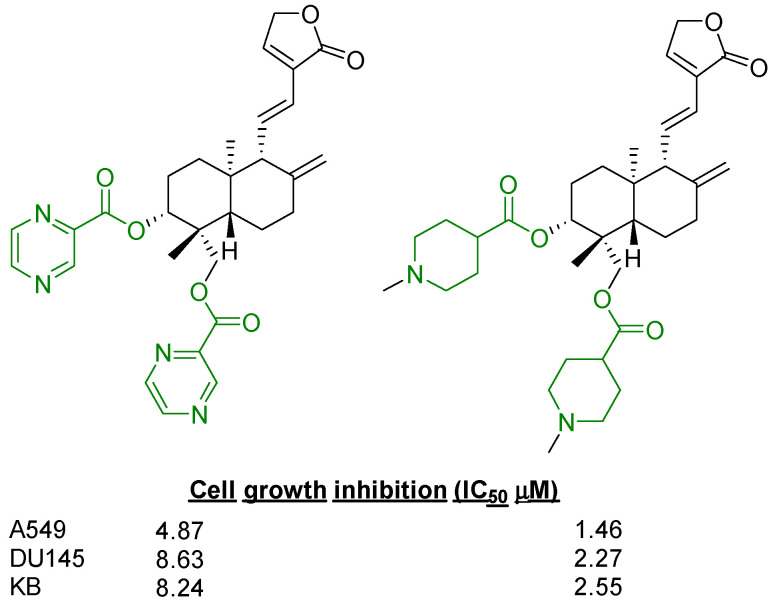
C3, C19 esters of andrographolide with anticancer activity.

C3 and C19 diesters of 14-deoxy-11,12-didehydroandrographolide were synthesized by Xiao et al. [39] using various aza-heterocycles. Among the synthesized compounds, the most active against lung (A549), prostate (DU145) and oral (KB) cancer cells are displayed in Figure 8.

**Figure 9:**
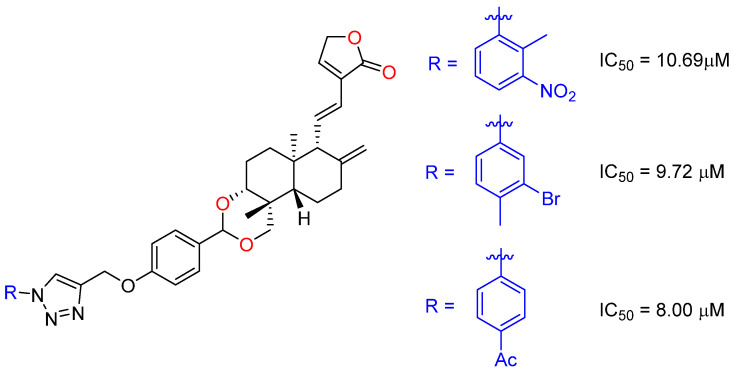
Anticancer activities of 1,2,3-triazole of 14-deoxydidehydroandrographolides.

The positive impact of 1,2,3-triazoles at position C14 of andrographolide, as well as at positions C3 and C19, was previously mentioned. Oh et al. synthesized 14-deoxy-11,12-didehydroandrographolide derivatives and tested them in various human cancer cell lines, including MCF-7 (breast cancer), MDA-MB-231 (triple-negative breast cancer), COLO205 (colon cancer), HepG2 (liver cancer), K562 (leukemia), HeLa (cervical cancer), HEK293 (human embryonic kidney) cells, to assess their cytotoxic activity. Contrary to expectations, most compounds showed selective anticancer activity against the K562 cell line, with IC50 values in the micromolar range, and significant cytotoxicity but were inactive in other cell lines [60] (Figure 9).

**Figure 10:**
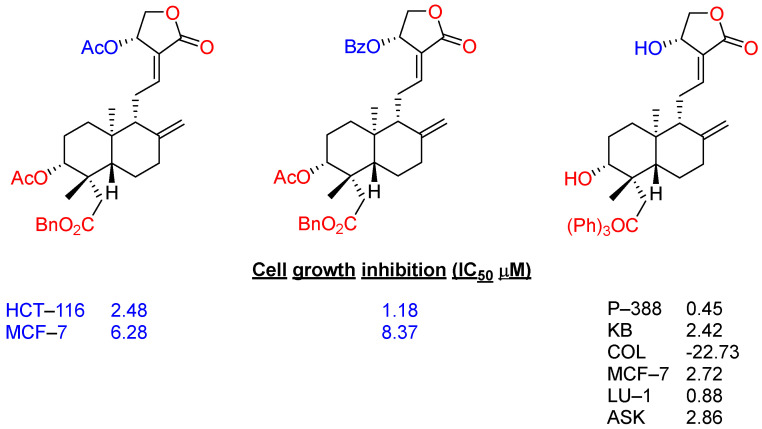
Effects of representative active andrographolide analogues in cancer cells.

A series of andrographolide-19-oic acids were synthesized by Chen et al. [61] and tested in vitro in two human cell lines, i.e., HCT-116 (colon cancer) and MCF-7 (breast cancer). The best compounds (Figure 10), with IC50 values in the range of 1–3 μM, displayed superior cytotoxicity compared to andrographolide. Similarly, the oxidative conversion of the C19 alcohol into a carboxyl group resulted in a significant increase in cytotoxicity [58]. Further studies on a number of analogues showed that a silyl ether or methyl ether triphenyl at C19 increased toxicity in cancer cells, including P-388, KB, COL-2, MCF -7, LU-1 and ASK cells, respectively involved in mouse leukemia, colon, breast and lung cancers. The 19-*O*-triphenyl methyl ether analogue even showed greater cytotoxic activity than the powerful anticancer drug ellipticine [62].

**Figure 11:**
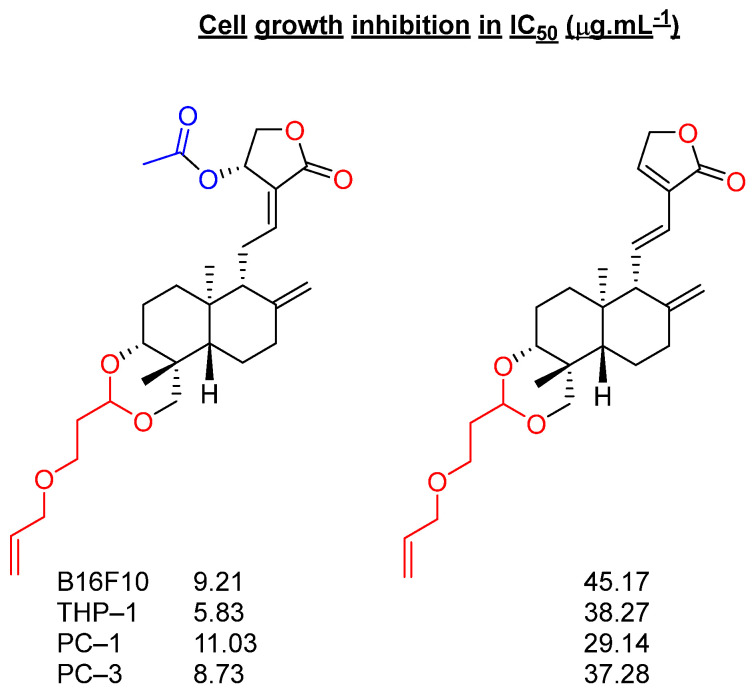
Cell growth inhibition of 3-(allyloxy)propylidene acetals of andrographolide.

The 14-acetoxyandrographolide and 14-deoxy-11,12-didehydroandrographolide derivatives, modified by acetalization of the hydroxyl functions at positions C3 and C19, showed very good cytotoxicity (IC50 < 10 μg/mL) in most of the cancer cell lines tested, i.e., B16F10, THP-1, PC-3 and SKOV3 (skin, leukemia, prostate and ovarian cancer cell lines, respectively) [63] (Figure 11).

#### 2.2.2. C14, C12, C3 and C19 Modifications

**Figure 12:**
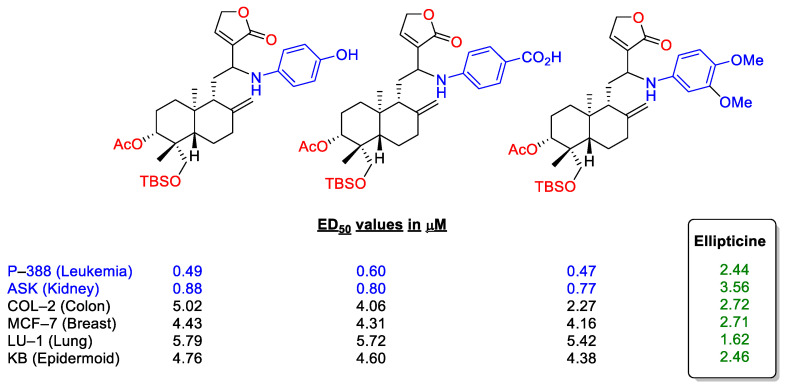
Andrographolide analogues nitrogen-substituted at the C12 position. ED_50_ values (drug concentration causing 50% of growth inhibition) in μM.

Kasemsuk et al. [64] generated a series of compounds substituted at C12 by arylamino derivatives. By removing the hydroxyl group at C14 and modifying those at C3 and C19, they generated a library of compounds, most of which showed cytotoxicity in several cancer cell lines. When the C12-grafted aniline was substituted at the para position, the cytotoxic activity increased in P-388 (leukemia) and ASK (Atlantic salmon kidney) cells. The selected derivatives (Figure 12) were found to be more potent than ellipticine [64].

**Figure 13:**
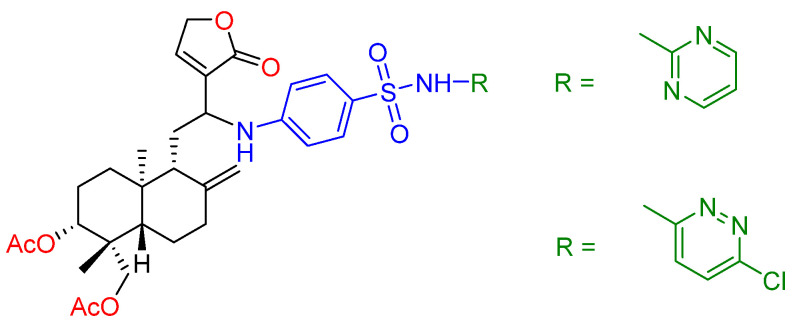
Sulfonamide-related andrographolide with anticancer activity.

Similarly, Golakoti et al. [65] modified 14-deoxy-andrographolide at the C12 position by introducing sulfonamide groups. Once again, the cytotoxicity (GI50, TGI and LC50) was found to be superior to that of andrographolide. The compounds were also non-toxic to normal human dermal fibroblast (NHDF) cells, with THZ-1 (a covalent CDK7 inhibitor) used as the reference drug [65] (Figure 13).

Among the C3, C19, C14 and C12-modified derivatives, 3,19-di-*O*-acetyl-12-phenylthio-14-deoxy-andrographolide emerged as one of the most potent compounds reported in 2015 by Kandanur et al. [66]. It exhibited cytotoxic activity against the HCT-116 cell line (GI50: 0.85 μM) and other cell lines. From the various studies cited, it appears that the anticancer activity of andrographolide and its derivatives is due to the induction of cell cycle arrest and apoptosis.

**Figure 14:**
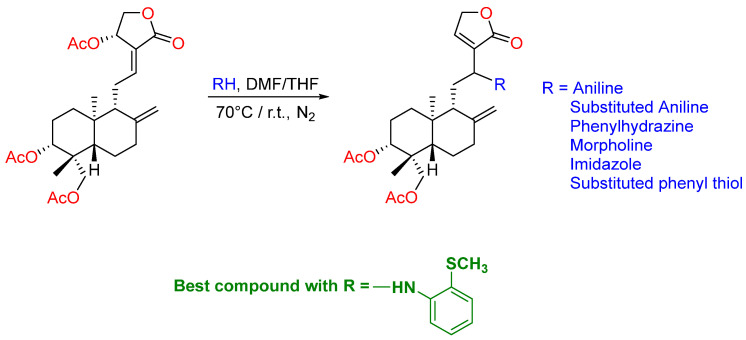
Preparation of 3,19-di-*O*-acetyl-C12-substituted-14-deoxyandrographolide derivatives.

To assess the potential for apoptosis induction in the HCT-116 cell line, Kandanur et al. synthesized an array of compounds bearing diverse substituents at the C12 position of 3,19-di-*O*-acetyl-C-12-substituted-14-deoxy-andrographolide [32]. The most active derivative had a (2-methylthio)phenylamino group at the C12 position (Figure 14).

New andrographolide dithiocarbamates were synthesized by Saeeng et al. [67]. These sulfur derivatives might have chemopreventive activities, particularly against breast cancer. Notably, sulfur functions are also found in natural products derived from cruciferous vegetables, such as brassinin, isolated from cabbage, whose derivatives showed potential chemopreventive activity against cancer [68]. Introducing this dithiocarbamate function is expected to enhance the cytotoxic efficacy of andrographolide.

**Scheme 6:**
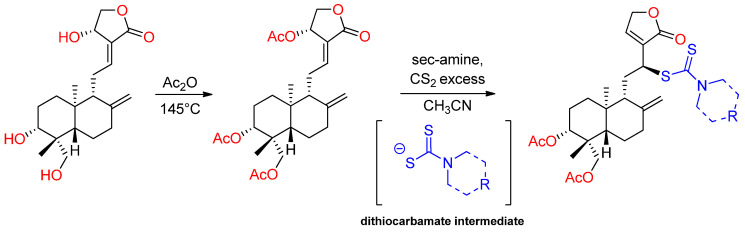
Synthesis of 12-dithiocarbamoyl-14-deoxyandrographolide analogues.

Saeeng et al. [67] introduced this function at the C12 position using tandem reactions followed by elimination, conducted in a one-pot process without catalysts and under mild conditions. The resulting series of 12-dithiocarbamoyl-14-deoxyandrographolide derivatives was tested on numerous cell lines: P-388 (murine leukemia), KB (oral carcinoma), HT-29 (colorectal), MCF-7 (breast), A-549 (lung), ASK (rat glioma), KKU-M213 (human intrahepatic cholangiocarcinoma), HuCCA-1 (human cholangiocarcinoma-derived cell line) and KKU-055 (cholangiocarcinoma, biliary tract). The cytotoxic activities of the different compounds were assessed using a sulforhodamine B (SRB) assay. Introducing morpholine dithiocarbamate at C12 significantly increased the cytotoxic activity in most cancer cell lines, up to 5-fold in HuCCA-1 cancer cells. The hypothesis was that this dithiocarbamate fragment provided a lipophilic character, enhancing the compound’s ability to cross cell membranes. While the introduction of the dithiocarbamate moiety increased the cytotoxic activity in some cases, particularly in MCF-7 and ASK cells, some products were inactive, and others showed stronger activity than the positive control ellipticine. Therefore, further development of libraries of hemi-synthetic analogues is needed to target specific pathologies [68] (Scheme 6).

**Scheme 7:**
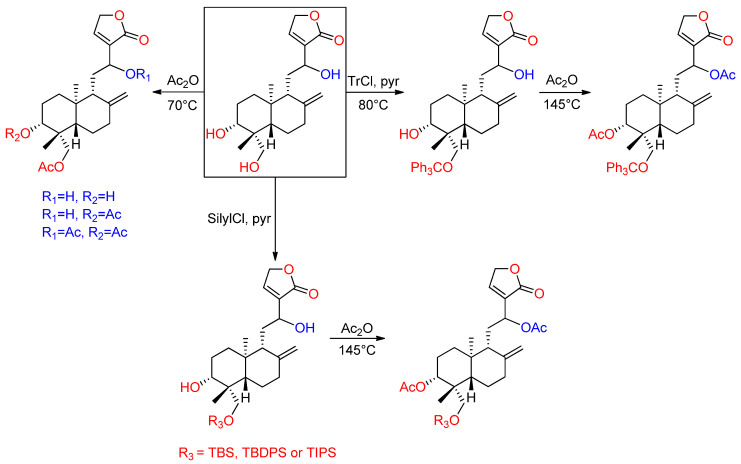
Synthesis of 14-deoxy-12-hydroxyandrographolide derivatives.

The introduction by Saeeng et al. [69] of a hydroxyl at C12 significantly improved the cytotoxic activity of andrographolide analogues. These analogues were evaluated in vitro on six cancer cell lines, i.e., P-388 (murine leukemia), KB (oral carcinoma), COL-2 (colon), MCF-7 (breast), LU-1 (lung) and ASK (breast), using the SRB assay, with ellipticine as a positive control. The cytotoxicity of 14-deoxy-12-hydroxyandrographolide derivatives, featuring various protective groups on the C3 and C19 hydroxyls, revealed a significant improvement (9- to 20-fold over that of andrographolide for many cell lines, except P-388) when a trityl or silyl ether group (TBDPS and TIPS) was located at C19. This substitution at C19 therefore seemed to play a crucial role in cytotoxic activity. Similarly, C8-C17 *exo*-epoxidation significantly increased the cytotoxicity. According to Saeeng’s findings, the cytotoxicity of these compounds seems to be more dependent on their lipophilicity than on the preservation of any specific fragment of the natural molecule (Scheme 7) [69].

#### 2.2.3. C14, C12, C8, C17, C3 and C19 Modifications

**Figure 15:**
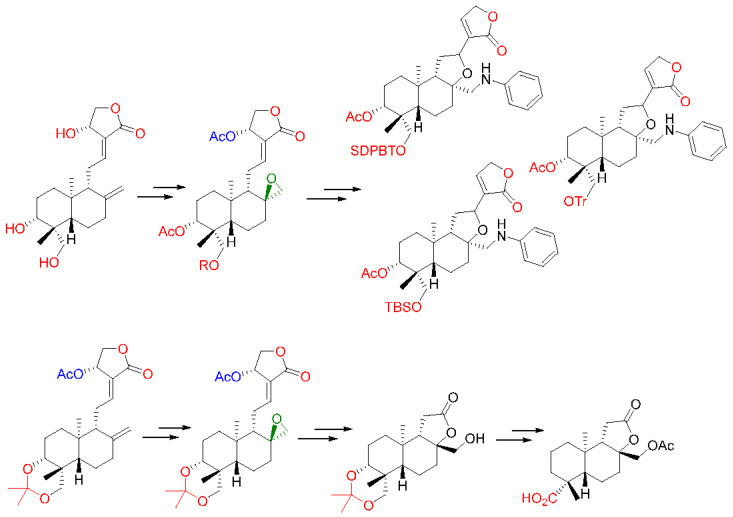
Isoandrographolide and *epi*-isoandrographolide analogues.

Kasemsuk et al. [70] investigated various *epi*-isoandrographolides for their anticancer activities. They modified andrographolide through a series of tandem reactions to synthesize a series of 17-amino-8-*epi*-isoandrographolide analogues (Figure 15). These derivatives showed strong activity against ASK cancer cells and exhibited selective cytotoxic activity in these cells [71]. However, the cytotoxic activity was moderate against A549 (lung cancer) and HeLa (cervical cancer) cell lines [71].

### 2.3. Modification at Positions C3 and C19

**Figure 16:**
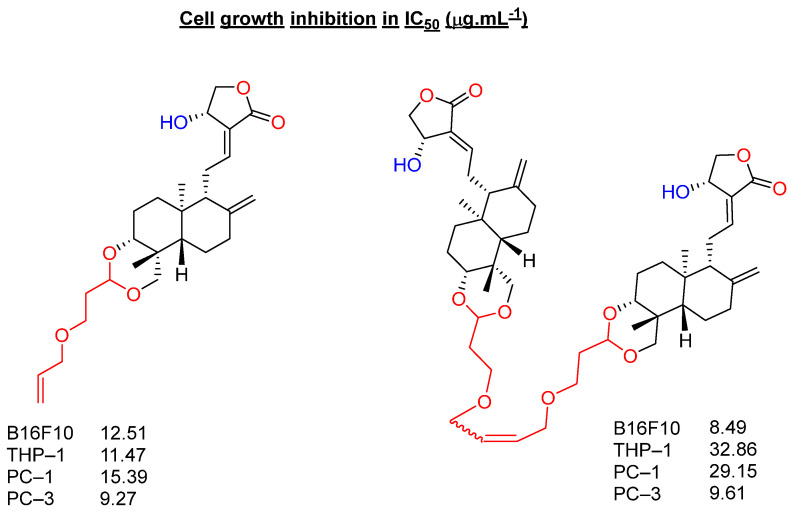
Cell growth inhibition of a 3-(allyloxy)-propylidene acetal of andrographolide and related dimeric compound.

Derivatives of andrographolide, modified by forming a 3-(allyloxy)-propylidene acetal on the hydroxyl functions at the positions C3 and C19, and their homodimeric compound obtained through metathesis, also showed excellent cytotoxicity (IC50 < 10 μg mL^−1^) in B16F10, THP-1, PC-3 and SKOV3 cancer cell lines (skin, leukemia, prostate and ovarian cancers, respectively, Figure 16) [63].

**Figure 17:**
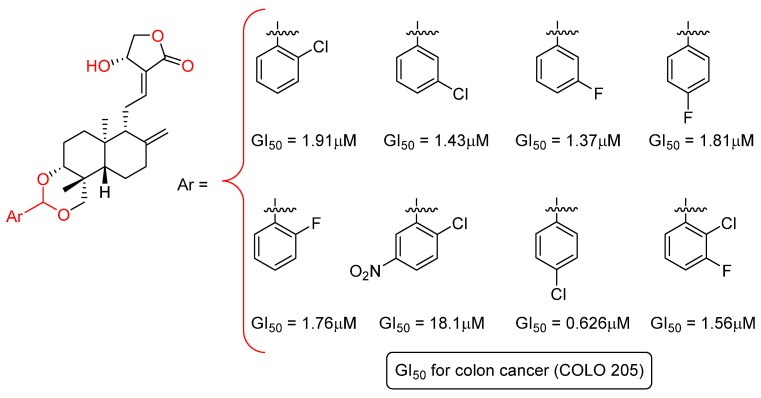
Andrographolide analogues with strongest activity against colon cancer.

Compounds modified by Stanslas et al. [72] at the C3, C19 positions via the formation of arylidene acetalic rings were screened on 60 NCI (National Cancer Institute, USA) human tumor cell lines derived from nine cancer cell types. Moderate to excellent cell growth inhibition results were recorded (Figure 17).

**Figure 18:**
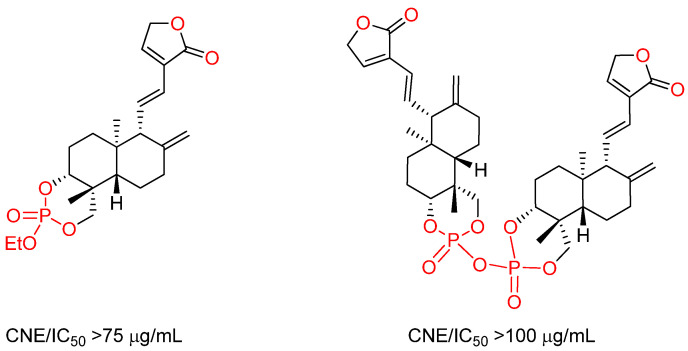
Cyclophosphorylated andrographolide derivatives with antitumor activity.

Wang et al. [73] also developed a series of andrographolides bearing a cyclophosphate group at positions C3, C19, as well as a dimeric pyrophosphate. These compounds were found to be highly effective for their antitumor activity in vitro (Figure 18).

**Figure 19:**
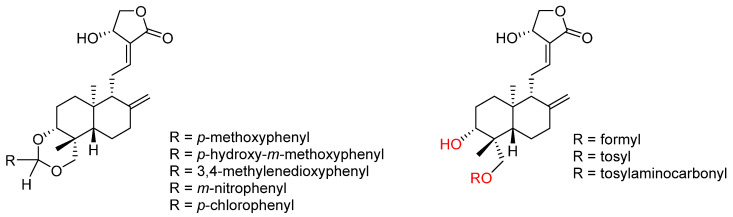
3,19-Acetals and 19-monoesters derived from andrographolide with antitumor activity.

Bath and Menon [74] obtained various compounds by hemisynthesis. Starting from andrographolide and reacting it with various substituted benzaldehydes under acid catalysis, 3,19-benzylidene acetals were prepared. Furthermore, selective esterification of the primary hydroxyl group at C19 was carried out to yield the corresponding formate, tosylate, and carbamate derivatives (Figure 19). The biological activity of those compounds was assessed in the A549 cell line (lung cancer) using the SRB assay, a sensitive measure of drug-induced cytotoxicity [75]. By measuring the percentage of cell growth inhibition, the authors demonstrated that derivatives protected at both C3 and C19 exhibited superior tumor activity in this cell line than those substituted only at C19.

### 2.4. Modification at Positions C8–C17

**Scheme 8:**
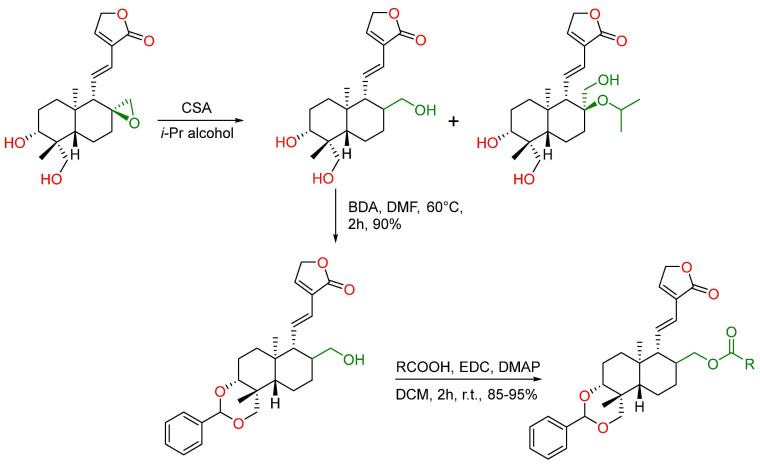
Synthesis of 3,19-benzylidene-14-deoxy-11,12-didehydroandrographolide C17 esters from the C8-C17 *exo*-epoxides.

In 2023, Kumar et al. synthesized a library of approximately 50 andrographolide analogues modified at the primary C17 position, with or without protection of the other hydroxyl groups [76]. This library underwent in vitro screening on four human cancer cell lines: A549 (lung), PC-3 (prostate), MCF-7 (breast) and HCT-116 (colon). Most of the analogues displayed a cytotoxic profile in all tested cells, compared to andrographolide itself. Among the derivatives synthesized (Scheme 8), the compounds bearing a *para*-methoxyphenylacetyl ester at the C17 position showed the highest efficacy. The 3,19-benzylidene acetal inhibited cell proliferation in the A549 (IC50: 6.6 μM) and PC-3 (IC50: 5.9 μM) cell lines, while the non-protected variant showed the best activity against the A549 cell line (IC50 of 3.5 μM). 

### 2.5. Other Modifications

**Scheme 9:**
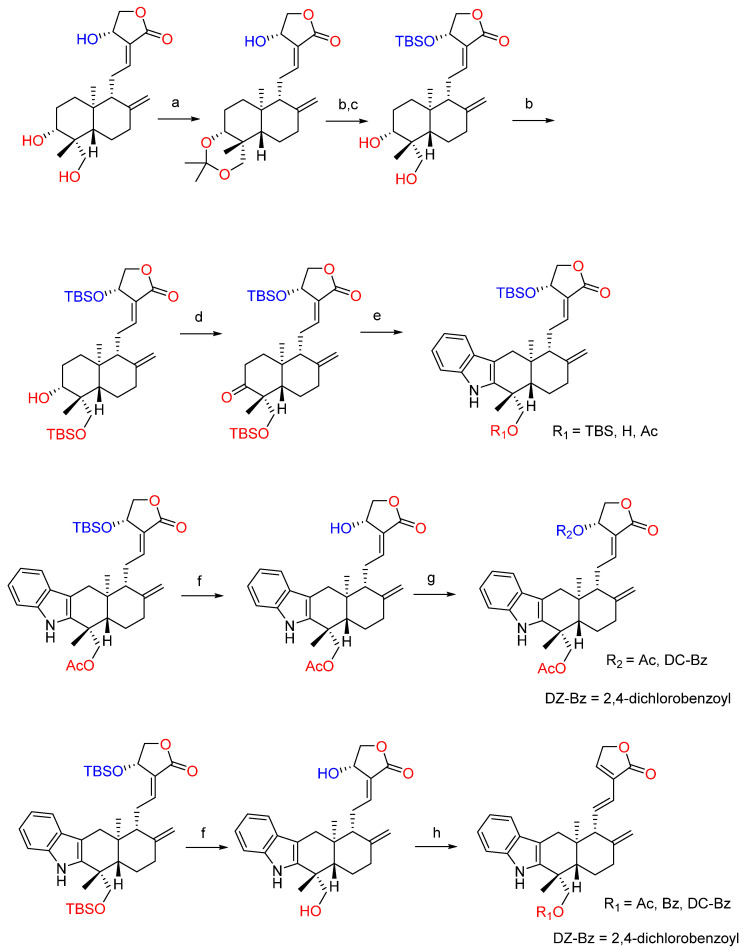
(**a**) 2,2-dimethoxypropane, p-TsOH (cat), Tol/DMSO, 80 °C, 93%. (**b**) TBSCl, imidazole; (**c**) AcOH/H_2_O: 7/3, r.t. (**d**) PCC; (**e**) PhNHNH_2_, HCl, AcOH; (**f**) TBAF, THF (**g**) anhydride (R_2_)_2_O or carboxylic acid (R_2_OH), EDCI, DMAP; (**h**) anhydride (R_1_)_2_O or carboxylic acid (R_1_OH), EDCI, DMAP.

Xue et al. conducted a major modification of the first six-membered ring of andrographolide [38], designing and preparing indolo[3,2-*b*]andrographolide derivatives via a Fischer indole synthesis (Scheme 9). The indolo analogues were subjected to in vitro screening using three human cancer cell lines, i.e., MCF7 (breast), HCT116 (colon) and DU145 (prostate). Several compounds exhibited greater activity than andrographolide, with one showing IC50 values of 1.85, 1.22 and 1.24 μM in MCF7, HCT116 and DU145 cells, respectively. This compound inhibited tumor proliferation through concentration-dependent cell apoptosis, resulting in cell cycle arrest (Scheme 9).

## 3. Conclusions

In the realm of cancer pathologies, andrographolide can undergo impactful modifications primarily at the hydroxyl functions, notably the lactone function, along with the C19 and C3 positions. Conversely, the epoxidation of the C8–C17 double bond does not consistently show significance, although it is challenging to establish a definitive rule, as epoxidation along with other modifications positioned elsewhere can enhance cytotoxicity. Position 12 underwent extensive alterations resulting in the formation of C-C, C-N, C-S and C-O bonds, showcasing substantial activity enhancements across numerous cell lines.

While modifications at C15 have been relatively neglected, recent attention has shed light on the benefits of incorporating variously substituted benzylidene moieties. Previously, the removal of C11–C12 double bonds and the lactone group was considered detrimental to the activity; however, recent findings suggest otherwise, yielding intriguing results. The landscape of potential pharmacomodulations remains vast, aiming to bolster the efficacy of this natural molecule and position it as a promising drug candidate. It is noteworthy that certain studies discussed here have shown that modifications can alter the molecule lipophilicity, which undoubtedly accounts for certain biological activities. Hence, it is important to measure this property when introducing functionalities during future pharmacomodulation studies of this molecule.

## Data Availability

Not applicable.
